# Inhibition of Tip60 Reduces Lytic and Latent Gene Expression of Kaposi’s Sarcoma-Associated Herpes Virus (KSHV) and Proliferation of KSHV-Infected Tumor Cells

**DOI:** 10.3389/fmicb.2018.00788

**Published:** 2018-04-24

**Authors:** Sydney Simpson, Guillaume Fiches, Maxime J. Jean, Michael Dieringer, James McGuinness, Sinu P. John, Meir Shamay, Prashant Desai, Jian Zhu, Netty G. Santoso

**Affiliations:** ^1^Department of Microbiology and Immunology, University of Rochester Medical Center, Rochester, NY, United States; ^2^Signaling Systems Unit, Laboratory of Systems Biology, National Institute of Allergy and Infectious Diseases, National Institutes of Health, Bethesda, MD, United States; ^3^The Azrieli Faculty of Medicine, Bar-Ilan University, Safed, Israel; ^4^Viral Oncology Program, Johns Hopkins School of Medicine, Johns Hopkins University, Baltimore, MD, United States; ^5^Department of Pathology, Ohio State University Wexner Medical Center, Columbus, OH, United States

**Keywords:** KSHV, HHV-8, Kaposi’s sarcoma, primary effusion lymphoma, Tip60, MG149, NU9056

## Abstract

Kaposi’s sarcoma-associated herpesvirus (KSHV) is an oncogenic virus responsible for the development of Kaposi’s sarcoma, primary effusion lymphoma (PEL), and Multicentric Castleman’s disease in immunocompromised individuals. Despite the burden of these diseases there are few treatment options for afflicted individuals, due in part to our limited understanding of virus-host interactions. Tip60, a histone aceytltransferase (HAT) has been previously shown to interact with both the KSHV latency associated nuclear antigen protein (LANA), which is the main factor in maintaining the viral latent state, and ORF36, a viral kinase expressed in the lytic phase. We further investigated Tip60-virus interaction to ascertain Tip60’s role in the viral life cycle and its potential as a target for future therapeutics. Through modulation of Tip60 expression in HEK293T cells harboring a plasmid containing the KSHV viral episome, Bac36, we found that Tip60 is vital for both lytic replication as well as efficient expression of latent genes. Interestingly, Tip60 small molecule inhibitors, MG149 and NU9056, similarly inhibited latent and lytic genes, and reduced virion production in wild-type KSHV^+^/EBV^-^ PEL, BCBL-1 cells. Long-term treatment with these Tip60 inhibitors selectively decreased the viability of KSHV-infected B lymphoma cells compared to uninfected cells. From this study, we conclude that Tip60 is important for KSHV infection and its associated cancer development, and Tip60 is therefore a potential target for future antiviral and anticancer therapeutics.

## Introduction

Kaposi’s sarcoma (KS) associated herpes virus (KSHV) also known as human herpes virus 8 (HHV-8) is a γ-herpes virus associated with KS endothelial lesions as well as several lymphoproliferative disorders, including primary effusion lymphoma (PEL) and Multicentric Castleman’s disease (Reddy and Mitsuyasu, 2011; Cesarman, 2014). These malignancies are most prevalent in immunocompromised individuals, particularly those infected with the human immunodeficiency virus (HIV) (Uldrick and Whitby, 2011). Although its incidence has declined with the adoption of highly active retroviral treatment (HAART), KS remains the most prevalent cancer in sub-Saharan Africa and the second most common cancer among AIDS patients (Gbabe et al., 2014). Currently, there is no standard treatment for KSHV-associated malignancies, aside from utilizing HAART in the context of HIV co-infection. Chemotherapy, antiviral drugs, and radiotherapy have been used to treat KS, but are toxic and not completely effective (Coen et al., 2014). Chemotherapy can be used to treat PEL, but PEL is generally resistant, resulting in a poor prognosis with a median survival period of 6 months (Okada et al., 2014). While there are presently no antiviral drugs licensed for KSHV-associated diseases, there is work being done to identify potential therapeutics, particularly viral DNA polymerase inhibitors. Additionally, antiviral drugs approved for use against other herpesviruses are being examined for their efficacy against KSHV (Sergerie and Boivin, 2003; Coen et al., 2014).

Similar to other herpesviruses, KSHV persists in the host cell as a life-long infection, forming a stable circular episome that tethers to the host chromatin (De Leon Vazquez and Kaye, 2011; Uppal et al., 2014). Once infected, host cells are usually unable to support the primary lytic replication of KSHV (Bechtel et al., 2003; Austgen et al., 2012; Dollery et al., 2014); thus, KSHV predominantly remains in a latent phase where it expresses only a few genes dedicated to episomal maintenance and immune evasion (Ganem, 2006). This quiescent latent phase primarily results from the expression of the latency associated nuclear antigen (LANA), which is responsible for tethering the episome to the host chromatin, as well as decreasing expression of the replication and transcription activator (RTA/ORF50) protein via binding to the RTA promoter (Lan et al., 2005). Nevertheless, KSHV can switch between latent and lytic gene expression resulting in the production of viral progeny.

During the lytic cycle a large array of viral genes are expressed in a regulated cascade, leading to the production and eventual release of newly synthesized viral particles. Lytic gene expression is divided into three distinct kinetic stages, with immediate early, early, and late gene transcripts expressed in a sequential manner (Gruffat et al., 2016). RTA is an immediate early lytic gene that is both necessary and sufficient for the induction of viral replication (Sun et al., 1998; Lukac et al., 1999; Gradoville et al., 2000). Immediate early genes upregulate early lytic genes. Early genes, such as K8 (K-bZip), a leucine zipper binding protein, are generally involved in DNA replication and late viral gene expression. Late lytic genes are expressed after DNA replication and typically encode viral structural proteins present on or used in virion assembly. Examples of late lytic genes include the viral capsid protein ORF26 and the glycoprotein K8.1, which shares an open reading frame with K8 and is derived by alternate splicing. Expression of the early gene K8 is induced first by the immediate early gene RTA, and then transactivates the late gene K8.1 (Seaman et al., 1999).

Host genes can modulate the lytic and latent replication of herpesviruses. Earlier studies identified that Tip60, a host cellular acetyltransferase, is phosphorylated by herpes-encoded protein kinases, including KSHV ORF36, and is required by another member of the γ-herpesviruse family, Epstein Barr virus (EBV), for efficient lytic replication (Li et al., 2011). Notably, LANA binds to Tip60 without affecting its stability (Shamay et al., 2012). Tip60 was originally discovered as a histone acetyltransferase (HAT), which interacts with the HIV Tat protein (Kamine et al., 1996). Tip60 is a transcriptional co-regulator utilized for either activation of tumor suppressors (Kim et al., 2005) or inactivation of oncogenes. It is also known to be involved in the DNA damage response (DDR), as Tip60 acetylates and activates ataxia-telangiectasia mutated (ATM), a key protein kinase involved in regulating the DDR (Sun et al., 2005; Li et al., 2011). Recently, KSHV infection has been shown to induce DDR, as well as utilize a number of proteins involved in this pathway, including ATM (Zhu et al., 2006; Singh et al., 2014; Hollingworth et al., 2015, 2017). In this study, we demonstrated that modulation of Tip60 expression in KSHV-infected HEK293T cells and Tip60 inhibition using small-molecular compounds in PEL cells, consistently resulted in decreased expression of viral lytic and latent genes, and reduced virion production. Additionally, we investigated whether Tip60 would be an effective target for the treatment of KSHV-related malignancies.

## Materials and Methods

### Cell Culture

HEK293T cells were maintained in 1x DMEM with 10% FBS and Penicillin-Streptomycin (100 U/ml and 100 μg/ml, respectively). BCBL-1 cells (Renne et al., 1996) were obtained from the NIH AIDS Reagents Program and maintained in RPMI with 10% FBS and 1% pen/strep. MC116.219 cells are an undifferentiated B lymphoma cell line MC116, infected with recombinant KSHV.r219 virus. These cells were kindly provided by Edward Berger at NIH (Vieira and O’Hearn, 2004; Cesarman, 2014; Dollery et al., 2014; Schulz and Cesarman, 2015). BJAB, BC-3, MC116, and MC116.219 cells were maintained in RPMI with 20% FBS and Penicillin-Streptomycin (100 U/ml and 100 μg/ml, respectively). All cells were culture at 37°C and 5% CO_2_. HEK293T.Bac36 cells harboring KSHV episomes were maintained with 100 μg/mL hygromycin. MC116.219 cells harboring KSHV episomes were maintained with 10 μg/mL puromycin.

### Lentiviruses

Viral particles for pAPM lentiviral vectors were prepared as previously described (Power et al., 2015). Briefly, HEK293T cells were co-transfected with psPAX2, PMD2.G (Addgene #12260, #12259), and the pAPM vector expressing NT shRNA 5′-CAC AAA CGC TCT CAT CGA CAA G-3′ or Tip60 shRNA 5′-TCG ATT TTC GCT TCC GTC CTG G 3′, using TurboFect^TM^ Transfection Reagent (Thermo Fisher) following the manufacturer’s protocol. The cell supernatant containing viral particles was harvested, passaged through a 0.45 μm filter (Sartorius), and stored at -80°C. HEK293T.Bac36 cells were transduced with lentiviral particles together with polybrene (1,5-Dimethyl-1,5-diazaundecamethrylene polymethobromide) at a 1:1000 ratio to facilitate viral entry. Cells were incubated for 3 days and then selected by adding puromycin (Life Technologies) into the medium at 1 μg/mL.

### Plasmid Transfection

HEK293T cells were transfected with the modified KSHV Bac36 genome containing the hygromycin resistance gene and the GFP gene as described previously (Zhou et al., 2002) using the TurboFect^TM^ Transfection Reagent (Thermo Fisher) following the manufacturer’s protocol. Cells were incubated for 3 days and then selected by adding hygromycin (Mirus Bio LCC) into the medium at 200 μg/ml. Similarly, pLX317 vector expressing V5-Tip60 was transfected into HEK 293T.Bac36 cells and selected with 3.5 ng/ml puromycin. pLX vectors were picked from the MISSION^®^ TRC3 Human LentiORF Collection Library (Millipore Sigma).

### Drug Treatment

Cells were pre-treated with Tip60 inhibitors, MG149 (Axon MedChem) or NU9056 (Tocris Bioscience), or general HAT inhibitors, curcumin (Santa Cruz Biotech) or garcinol (Santa Cruz Biotech), for 6 h. KSHV reactivation was induced by treating HEK293T.Bac36 cells with 20 ng/mL 12-O-Tetradecanoylphorbol-13-acetate (TPA) (Sigma-Aldrich) and 1.5 mM sodium butyrate (SB) (Sigma-Aldrich). For MC116.219, BCBL-1, and BC-3 cells, 0.3 mM of SB was used instead.

### Reverse Transcription (RT) and Quantitative Real-Time PCR (qPCR)

Total mRNA was extracted from cells using the RNeasy Mini Kit (Qiagen) and then reverse transcribed into cDNA using the iScript cDNA Synthesis Kit (Bio-Rad) according to the manufacture’s instructions. The cDNAs were subjected to real-time qualitative PCR (RT-qPCR) analysis using the iTaq Universal SYBR Green Supermix (Bio-Rad) and the primer sets targeting the investigated genes on the CFX Connect^TM^ Real Time System (Bio-Rad). The primer sequences were as follows: 5′- GCC TCT TGT CTC TTA GAT TTG GTC -3′ and 5′- TAG CAC TCA CCA TGT AGT TGA GGT -3′ for GAPDH, 5′- TCT ACC TGT GCG AGT TCT GC -3′ and 5′- CCC TTG CGG TAA ATC TCA TT -3′ for Tip60, 5′-CCT GGA CGC TCT CTC ACA CA -3′ and 5′- GGA TCT GCG AGT TGG AAG CT -3′ for K8, 5′- CCT TCG GCC CGG GGT CT -3′ and 5′- CGG TGG CAG TTG CGT ATA CTC T -3′ for ORF50, 5′- AGC CGA AAG GAT TCC ACC AT -3′ and 5′- TCC GTG TTG TCT ACG TCC AG -3′ for ORF26, 5′- TTA CCT CCA CCG GCA CTC TT -3′ and 5′ -GGA TGG GAT GGA GGG ATT G -3′ for LANA, 5′- CAT TGC CCG CCT CTA TTA TCA -3′ and 5′- TGA CGT TGG CAG GAA CCA-3′ for vCyclin, and 5′- GGA GGA GGG CAG GTT AAC GT-3′ and 5′- TGC GAC CTG CAC GAA ACA-3′ for vFLIP.

### Measurement of Viral DNA and Virus Production

Genomic DNA was isolated from cells using a DNeasy kit (Qiagen). Relative genome copy number was measured by qPCR for LANA and normalized to cellular GAPDH DNA levels.

Cells were stimulated with TPA/SB for 72 h with or without prior drug treatment as described above. Culture supernatants were centrifuged to remove cellular debris then passaged through a 0.45 μm filter. Filtered supernatants from HEK293T.Bac36 cell lines were used to infect HEK293T cells. Green fluorescent protein (GFP) expression was measured after 48 h using a BD Accuri^TM^ C6 Plus Flow Cytometer. Flow cytometry data were analyzed with FlowJo software (Version 10.0.8).

Virus production in PEL cell lines was measured as described previously (Watanabe et al., 2014; Nishimura et al., 2017). Briefly, virions were isolated as described above. In order to obtain only enveloped or encapsulated viral genomes the supernatant was treated with DNase I (Invitrogen). Viral DNA was then purified and extracted using a QIAamp Blood Mini Kit (Qiagen). Viral DNA isolated from virions was quantified by measuring LANA via qPCR.

### Virion Purification and Ultracentrifugation

BCBL-1 cells were pre-treated with Tip60 inhibitors for 6 h and then stimulated with 20 ng/mL TPA and 0.3 mM SB to reactivate KSHV. At 72 h post induction, cells were harvested and spun down by centrifuging twice at 400 ×*g* for 20 min. The supernatant was aspirated and passaged through a 0.45 μm filter, then placed over a 25% sucrose gradient and ultracentrifuged at 25,000 rpm at 4°C on a Beckman Coulter SW41Ti rotor for 1 h. The supernatant was removed and the virion pellet was resuspended in PBS to measure KSHV glycoprotein K8.1 present in the virions.

### Immunoblotting

Cells were harvested and lysed in 1x RIPA buffer (50 mM Tris-HCL [pH 7.4], 150 mM NaCl, 1% NP-40, 0.25% deoxycholic acid, and 1 mM EDTA). Cell lysates were sonicated at 40% power output for 1 min with 1 s pulses using a Fischer Scientific Model 120 Sonic Dismembrator. Virions from the supernatant were purified and resuspended in 1x PBS as described above. 4x LDS buffer (Thermo Scientific) and Beta-Mercaptoethanol (Acros Organics) at a ratio of 1:50 were added to cell or virion pellets for preparation of protein samples. Protein samples were boiled at 100°C for 5–10 min and then transferred to ice. Protein samples were further separated on a 4–20% tris-glycine gel (Novex, Life technologies). Proteins were transferred from the gel to a polyvinylidene fluoride (PVDF) membrane using an iBLOT2 dry transfer system (Life technologies). The primary antibodies used for immunoblotting, included anti-Tip60 (C-7) (Santa Cruz Biotech), anti-V5 (Invitrogen), anti-RTA/ORF50 (Abbiotec), anti-GAPDH (Santa Cruz Biotech), anti-K8.1 (4A4) (Santa Cruz Biotech), and anti-LANA (LNA-1) (Advanced Biotechnologies Inc.). Uncropped immunoblot images are available in Supplementary Data Sheet 2.

### Viability Assay

The effect of Tip60 inhibitors on cell viability and proliferation was measured over the course of 6 days. Specifically, on day 0 cells were seeded at a concentration of 37,250 cells/mL and treated with 5 μM MG149, 0.5 μM NU9056, or DMSO. Intracellular ATP levels were measured on days 2, 4, and 6 using the CellTiter-Glo^®^ Luminescent Cell Viability Assay (Promega) following the manufacturer’s protocol. Fresh inhibitors were added on days 2 and 4. Samples were aliquoted in triplicates on a 96-well plate, and the luminescence signal was measured using a Cytation^TM^ 5 plate reader (BioTek).

### Statistical Analysis

Significance for the differences between control and experimental groups was determined using a 2-tailed student’s *t*-test (Excel 8.0). *P*-values of 0.05 or 0.01 were considered significant and highly significant, respectively.

## Results

### Tip60 Promotes Efficient KSHV Viral Lytic Replication

**FIGURE 1:**
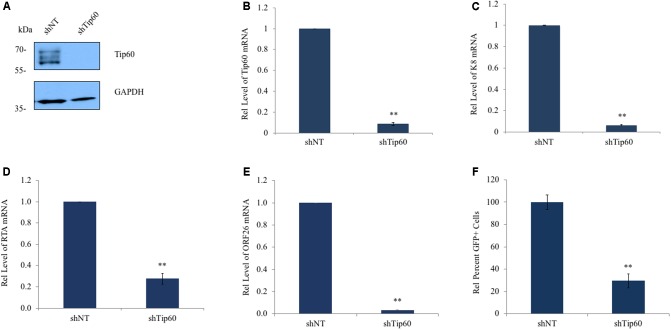
Tip60 promotes KSHV lytic gene expression. HEK293T.Bac36 cells were stably transduced with shRNA targeting Tip60 (shTip60) or non-targeting control (shNT) in pAPM vector. **(A)** Knockdown of Tip60 was confirmed by immunoblotting. GAPDH was used as a loading control. **(B)** Tip60 mRNA was measured by RT-qPCR, and normalized to shNT-expressing cells. **(C)** HEK293T.Bac36 cells that expressed shTip60 or shNT were treated with 12-O-Tetradecanoylphorbol-13-acetate and sodium butyrate (TPA/SB) for 48 h. The mRNA level of KSHV K8 lytic gene was measured by RT-qPCR and normalized to shNT-expressing cells. **(D)** RTA and **(E)** ORF26 mRNA levels were also measured. **(F)** 293T.Bac36 shNT and shTip60 cells were induced with TPA/SB. Supernatants were filtered and used to infect 293T cells. 48 h later GFP+ cells were counted using flow cytometry, and normalized to the average percent of GFP+ cells from shNT supernatants. RT-qPCR values were normalized to GAPDH. The results from three independent experiments are represented as mean ± SEM ^∗^*p* < 0.05, *^∗∗^p* < 0.01, student *t*-test.

To determine if Tip60 is involved in KSHV replication we knocked down Tip60 expression in HEK293T.Bac36 cells which were prepared by stably transfecting HEK 293T cells with the KSHV plasmid Bac36, as previously described (Zhou et al., 2002). These cells were transduced with a pAPM lentiviral vector expressing Tip60 shRNA (shTip60). A non-targeting shRNA (shNT) was used as a negative control. Expression of shTip60 in HEK293T.Bac36 cells significantly reduced endogenous Tip60 protein levels compared to shNT (**Figure 1A**). We also confirmed this knockdown effect by measuring Tip60 mRNA via qPCR (**Figure 1B**). HEK293T.Bac36 cells expressing shTip60 or shNT were treated with 12-O-Tetradecanoylphorbol-13-acetate (TPA, 20 ng/mL) and sodium butyrate (SB, 1.5 mM) to induce the lytic replication of KSHV. At 48 h post induction, we measured the mRNA levels of a KSHV lytic genes using qPCR. Knockdown of Tip60 significantly decreased K8 mRNA (**Figure 1C**). We further determined whether Tip60 is involved in KSHV lytic replication upstream of K8, through the KSHV replication and transcription activator, RTA. As with K8, knockdown of Tip60 drastically decreased RTA mRNA expression (**Figure 1D**). mRNA expression of ORF26, a late lytic gene encoding a viral capsid protein was also significantly decreased in these cells (**Figure 1E**). Interestingly, we found that this decrease in lytic gene transcription was not caused by a decrease in copies of the viral genome following Tip60 knockdown (Supplementary Figure S1). We next assessed virus production to further confirm that Tip60 promotes lytic replication. HEK293T.Bac36 cells expressing either shNT or shTip60 were induced for 72 h. Culture supernatants were harvested, filtered, and used to infect HEK293T cells. After 48 h, GFP+ cells were counted by flow cytometry. Supernatants from shTip60 expressing cells yielded significantly fewer GFP+ cells compared to shNT supernatants (**Figure 1F**), correlating with a 70% decrease in infectious virus production.

**FIGURE 2:**
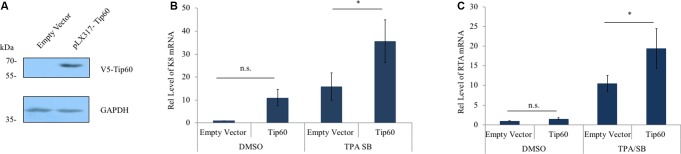
Tip60 overexpression increases early lytic gene expression. **(A)** HEK293T.Bac36 cells were stably transduced with V5-Tip60 in pLX317 or the empty vector. Expression of V5-Tip60 was confirmed by immunoblotting using an anti-V5 antibody. GAPDH was immunoblotted as a loading control. **(B)** HEK293T.Bac36 cells transduced with V5-Tip60 or empty vector were treated with TPA/SB or DMSO for 48 h. The mRNAs level of KSHV K8, and **(C)** RTA lytic genes were measured by RT-qPCR and normalized to DMSO-treated cells transduced with empty vector. RT-qPCR values were normalized to GAPDH mRNA. The results from three independent experiments are represented as mean ± SEM *^∗^p* < 0.05, *^∗∗^p* < 0.01, student *t*-test; n.s., non-specific.

To further validate our observations we determined the effect of Tip60 overexpression on KSHV early and immediate early lytic gene expression. A V5-tagged Tip60 cDNA was cloned into the pLX317 lentiviral vector. Either the pLX317-Tip60 or the empty pLX317 vector was stably transduced into the HEK293T.Bac36 cells. Overexpression of exogenous Tip60 was confirmed by immunoblotting using an anti-V5 antibody (**Figure 2A**). The mRNA levels of both K8 and RTA increased in response to Tip60 overexpression in TPA/SB-induced cells (**Figures 2B,C**). Interestingly, we also observed a slight increase in basal levels of K8 and RTA. These data support that Tip60 promotes KSHV lytic replication.

### Tip60-Specific Inhibitors Block Lytic Replication of KSHV

**FIGURE 3:**
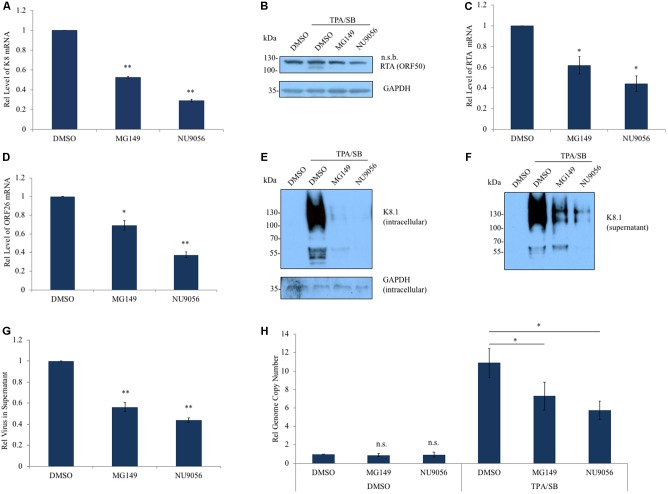
Inhibition of Tip60 reduces KSHV lytic gene expression. **(A)** BCBL-1 cells were pre-treated with a Tip60 inhibitor, MG149 (5 μM) or NU9056 (0.5 μM), for 6 h prior to stimulation with TPA/SB for 48 h to induce KSHV reactivation. mRNA level of KSHV K8 lytic gene was measured by RT-qPCR. **(B)** RTA expression was measured by immunoblot using an anti-RTA antibody; n.s.b., non-specific band. GAPDH was immunoblotted as a loading control. Both **(C)** RTA and **(D)** ORF26 transcription was measured using RT-qPCR. **(E)** KSHV glycoprotein K8.1 in cell lysates and in **(F)** viral pellets from ultracentrifuged cell free supernatant, 72 h post induction. **(G)** RT-qPCR measuring LANA was performed to measure the relative amount of virus in cell-free supernatant from BCBL-1 cells. **(H)** Relative amounts of viral DNA in BCBL-1 cells treated with DMSO or TPA/SB were measured using RT-qPCR 48 h post induction. For RT-qPCR experiments, aside from measuring virion DNA in the supernatant, values were normalized to GAPDH mRNA. The results from three independent experiments are represented as mean ± SEM ^∗^*p* < 0.05, ^∗∗^*p* < 0.01, student *t*-test.

We further tested the effect of Tip60 inhibition on KSHV lytic replication using two Tip60-specific HAT inhibitors (HATis), MG149 and NU9056, in BCBL-1 and MC116.219 cells. MG149 and NU9056 competitively bind to the Tip60 Acetyl-CoA binding and active thiol sites, respectively (Coffey et al., 2012; Ghizzoni et al., 2012). BCBL-1 is a PEL cell line that harbors latently infected KSHV (Renne et al., 1996). MC116.219 is an undifferentiated B lymphoma cell line (MC116) that is stably infected with the recombinant KSHV.r219 genome (Dollery et al., 2014). We titrated the drug cytotoxicity of Tip60 inhibitors (MG149 and NU9056) on both BCBL-1 and MC116.219 cells by measuring cell viability (Supplementary Figures S2A,B). Non-toxic concentrations of Tip60 inhibitors were used for all subsequent experiments (MG149, 5 μM; NU9056, 0.5 μM). BCBL-1 cells were pre-treated with MG149, NU9056, or DMSO (vehicle control) for 6 h and then stimulated with TPA and SB as previously described (Yu et al., 1999). Both Tip60 inhibitors significantly decreased K8 gene expression (**Figure 3A**). Similarly, NU9056 decreased K8 expression in a dose dependent manner in MC116.219 cells (Supplementary Figure S2C). In order to confirm that this effect is specific to Tip60 inhibition and not caused by general HAT inhibition, we also tested whether broadly acting HATis similarly reduced KSHV lytic gene expression. We used curcumin and garcinol, which inhibit 5-LO/Cox-2/NOS2 and p300/PCAF, respectively (Balasubramanyam et al., 2004; Marcu et al., 2006). As with the Tip60 inhibitors, we titrated the drug cytotoxicity of these general HATis (Supplementary Figures S2D,E). Treatment with curcumin (1μM) or garcinol (0.5 μM) did not reduce the mRNA level of K8 in TPA/SB stimulated BCBL-1 (Supplementary Figure S2F) or MC116.219 cells (Supplementary Figure S2G).

We next investigated the effect of Tip60 inhibition on immediate early and late gene expression. Tip60 inhibitor treatment reduced RTA protein and mRNA in TPA/SB induced BCBL-1 cells (**Figures 3B,C**). ORF26 transcription was also significantly decreased in these cells (**Figure 3D**). Additionally, both RTA and ORF26 transcription were decreased in TPA/SB stimulated MC116.219 cells titrated with NU9056 (Supplementary Figures S2H,I). We also determined the effect of Tip60 inhibitors on the protein expression of another KSHV late lytic gene, the viral glycoprotein K8.1. BCBL-1 cells were pre-treated with MG149 or NU9056, and stimulated with TPA/SB. We harvested both the cells and supernatants at 72 h post reactivation, and KSHV virions in the supernatant were concentrated using ultracentrifugation. The protein level of K8.1 present in the cellular lysate and in concentrated virions was measured by immunoblotting. Tip60 inhibition significantly reduced K8.1 protein in both cells and supernatants, indicative of a decrease in virus production (**Figures 3E,F**).

To confirm that Tip60 inhibitor treatment decreased virus production we extracted DNA from cell-free supernatants and cell lysates of TPA/SB stimulated cells. BCBL-1 cells were treated with Tip60 inhibitors and reactivated as before. The supernatants were harvested and cells and debris were removed centrifugation. In order to obtain only encapsulated and enveloped viral genomic DNA, the supernatants were filtered and then treated with DNase I. Virion DNA was extracted and the relative amount of virus was quantified using qPCR. Tip60 inhibitor treatment significantly decreased virion production (**Figure 3G**). Finally, we measured the levels of viral genomic DNA following Tip60 inhibitor treatment in both uninduced and induced BCBL-1 cells. As with the 293T.Bac36 cells (Supplementary Figure S1), Tip60 inhibition did not result in a loss of viral genome copies in latent cells. However, in TPA/SB stimulated cells MG149 and NU9056 treatments reduced viral genomes by 33 and 48%, respectively, indicating that inhibiting Tip60 blocks lytic DNA replication (**Figure 3H**). These results suggest that Tip60 is critical for expression of all three types of KSHV lytic genes (immediate early, early, and late), and that Tip60 inhibition using small-molecule compounds can potently affect KSHV lytic replication.

To further validate that Tip60 inhibition reduces productive lytic replication in KSHV-infected PEL cells, we tested the effect of our Tip60 inhibitors on BC-3 cells, another KSHV-infected PEL cell line. Tip60 inhibitor treatment significantly reduced K8, RTA, and ORF26 mRNA levels in TPA/SB-stimulated BC-3 cells (Supplementary Figures S3A–C), although the effects were not as strong as those observed in the BCBL-1 cells (**Figure 3**). Similarly, Tip60 inhibitor treatment reduced virus production in these cells ∼50% (Supplementary Figure S3D).

### Tip60 Regulates Expression of KSHV Latent Genes

**FIGURE 4:**
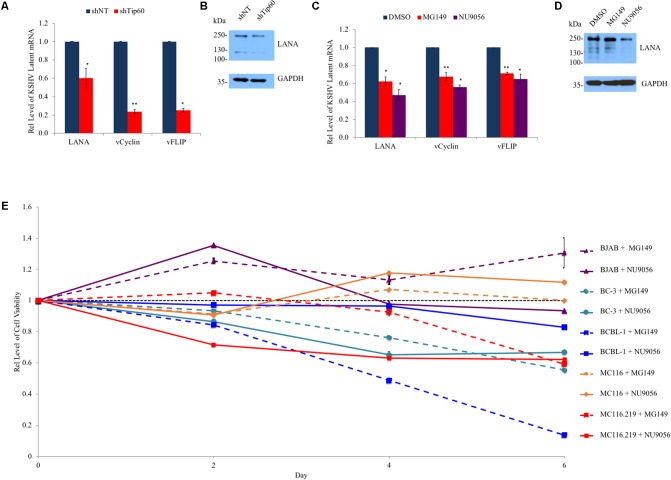
Role of Tip60 in KSHV latent gene expression and proliferation of KSHV-infected tumor cells. **(A)** HEK293T.Bac36 cells were stably transduced with shTip60 or shNT in pAPM vector. mRNA levels of KSHV latent genes (LANA, vCyclin, and vFlip) were measured by RT-qPCR and normalized to shNT-expressing cells. **(B)** Immunoblotting was used to measure LANA protein levels. **(C)** BCBL-1 cells were treated with Tip60 inhibitors MG149 (5 μM), NU9056 (0.5 μM), or DMSO for 48 h. mRNA levels of KSHV latent genes (LANA, vCyclin, and vFlip) were measured by RT-qPCR and normalized to DMSO-treated cells. **(D)** BCBL-1 LANA protein levels were measured by immunoblotting. **(E)** KSHV-positive PEL B cell lines (BC-3, BCBL-1, and MC116.219) and KSHV-negative cell lines (BJAB and MC116) were treated with MG149 (5 μM) or NU9056 (0.5 μM) for 6 days. Cell viability of these cells was measured every other day and normalized to DMSO-treated cells (black). For RT-qPCR values were normalized to GAPDH mRNA. For immunoblots GAPDH was used as a loading control. The results from three independent experiments are represented as mean ± SEM. ^∗^*p* < 0.05, ^∗∗^*p* < 0.01, student *t*-test.

During infection, particularly in the case of PEL, KSHV primarily remains in the latent phase (Judde et al., 2000; Speck and Ganem, 2010; Cesarman, 2014) with very few cells spontaneously entering the lytic cycle at any time. Latent genes function in immune evasion and contribute to viral oncogenesis and cell proliferation (Cesarman, 2014). Given that Tip60 plays a role in both cancer development and KSHV lytic replication, we investigated whether Tip60 is required for the expression of KSHV latent genes at the quiescent stage. We first determined the impact of Tip60 knockdown on the expression of a set of KSHV latent genes (LANA, vCyclin, and vFLIP) in HEK293T cells stably expressing shTip60 or shNT. Tip60 knockdown significantly reduced the mRNA levels of all tested KSHV latent genes, with a stronger suppressive effect on vCyclin and vFlip expression (∼75% decrease) compared to LANA expression (∼40% decrease) (**Figure 4A**). However, LANA protein levels were decreased in these cells (**Figure 4B**).

Next, we determined the impact of Tip60 inhibitors on the expression of the above KSHV latent genes in PEL cells. Treatment of BCBL-1 cells with Tip60 inhibitors (MG149, 5 μM; NU9056, 0.5 μM) for 48 h moderately reduced the expression of all three KSHV latent genes (LANA, vCyclin, vFLIP) by 30–50%, compared to DMSO treatment (**Figure 4C**). An immunoblot confirmed that Tip60 inhibitor treatment reduced LANA protein levels (**Figure 4D**). BC-3 cells were also treated with Tip60 inhibitors; however, this did not result in a detectable change in latent gene expression (data not shown).

### Tip60 Inhibitors Preferentially Block Proliferation of KSHV-Infected Tumor Cells

Since Tip60 inhibitors reduced the expression of KSHV latent oncogenic genes, we expected that the long-term treatment of KSHV-positive tumor cells with these compounds would impair cell proliferation. KSHV-positive B lymphoma cell lines (BCBL-1, BC-3, and MC116.219), as well as KSHV-negative cell lines (BJAB and MC116), were treated with DMSO, MG149 (5μM), or NU9056 (0.5 μM) to measure effects on cell viability. Cells were continuously cultured in the presence of these compounds for up to 6 days, and a fraction of cells was removed from culture for a cell viability assay every 2 days. We added Tip60 inhibitors into the culture at the same concentrations every 2 days. The results from MG149 or NU9056 treated cells were normalized to that of DMSO-treated cells for each cell line. Interestingly, KSHV-infected cells (BCBL-1, BC-3, and MC116.219) were more susceptible to cell cytotoxicity from at least one of the Tip60 inhibitors compared to KSHV-negative cells (BJAB and MC116), especially at day 6 (**Figure 4E**). These results clearly demonstrate that Tip60 inhibitors preferentially reduce cell proliferation of KSHV-positive tumor cells, indicative of their potential to selectively treat KSHV-associate malignancies.

## Discussion

The role of Tip60 in herpesvirus infection is conserved across all members of this viral family (Li et al., 2011). The virus-encoded kinase BGLF4 of Epstein Barr Virus (EBV) phosphorylates and activates Tip60, triggering the DDR pathway and facilitating viral lytic replication (Li et al., 2011). However, while several KSHV proteins (LANA and ORF36) have been implicated to interact with Tip60, the exact contribution of these interactions has yet to be determined (Li et al., 2011; Shamay et al., 2012). In this study, we investigated the role of Tip60 in regulating the life cycle of KSHV as well as the antiviral and anticancer potential of Tip60 inhibitors for treating KSHV-related malignancies. Through multiple experimental approaches, including loss- or gain-of-function analyses and use of Tip60-specific inhibitors, we discovered that Tip60 promotes both lytic and latent gene expression of KSHV (**Figures 1–4**). Intriguingly, B lymphoma cell lines harboring a latent KSHV infection were more susceptible to treatment with Tip60 inhibitors, with respect to cell viability, compared to uninfected cell lines (**Figure 4E**). These results suggest that Tip60 is critical for KSHV infection and could be targeted using small-molecule compounds to eliminate KSHV-positive tumor cells. This could also be true in the context of EBV-associated cancers since previous studies already confirmed that Tip60 is critical for EBV replication (Li et al., 2011).

Similar to what is observed with EBV, Tip60 knockdown by RNAi in HEK293T.Bac36 decreased early, immediate early, and late (K8, RTA, and ORF26) lytic gene expression. Consistently, overexpression of Tip60 increased both K8 and RTA expression. In KSHV-positive PEL cell lines BCBL-1 and BC-3, treatment with Tip60 inhibitors similarly reduced the expression of all three classes of lytic genes. Notably, inhibition of Tip60 in both HEK293T.Bac36 and PEL cell models decreased virus production. Importantly, these decreases in gene expression and virus production were not due to a decrease in viral genome copies following Tip60 inhibition. In latency, KSHV relies on the host cellular machinery to maintain viral copy numbers through a latent origin of replication. However, during lytic replication the virus utilizes duplicated origins of lytic replication, along with its own DNA polymerases and accessory factors, including RTA and K8, to substantially increase viral copies for virion production (AuCoin et al., 2002; Lin et al., 2003; Wang et al., 2006; Aneja and Yuan, 2017). While Tip60 inhibition did not change viral copies in latent cells, we did observe decreased viral genomic DNA in TPA/SB stimulated BCBL-1 cells treated with Tip60 inhibitors compared to DMSO (**Figure 3H**). These results could be interpreted in two ways: (1) Tip60 is only used for the induction of RTA/ORF50 (immediate early), the viral transactivator, which further turns on the lytic cascade (K8, early; ORF26 and K8.1, late) or (2) Tip60 is utilized for expression of all of these lytic genes through the association with their individual promoters. These possibilities will be the subject of further investigation.

We further confirmed that Tip60 promotes the expression of KSHV latent genes (LANA, vCylcin, vFLIP) in both HEK293T.Bac36 and BCBL-1 cells. This could be relevant to KSHV-induced oncogenesis, as previous studies have shown that KSHV latent genes possess oncogenic potential (Fakhari et al., 2006; Speck and Ganem, 2010; Uldrick and Whitby, 2011; Sin and Dittmer, 2013; Uppal et al., 2014; Schulz and Cesarman, 2015; Purushothaman et al., 2016). For example, transgenic mice whose B-cells express either LANA or the entire KSHV latent gene locus develop malignancies similar to KSHV-associated lymphomas (Fakhari et al., 2006; Sin and Dittmer, 2013). Our results reveal that Tip60 promotes expression of genes at the KSHV latency locus, and that Tip60 inhibitors can reduce the expression of these genes. Interestingly, we observed that the expression of vCyclin and vFLIP decreased more significantly than LANA in shTip60-expressing HEK293T.Bac36 cells (**Figure 4A**). While all three genes are co-transcribed from the LANA promoter, vCyclin and vFLIP are additionally transcribed from the kaposin promoter (Talbot et al., 1999; Pearce et al., 2005). Thus, Tip60 may also associate with the kaposin promoter beyond the LANA promoter, which places another layer of regulation on vCyclin and vFLIP expression. However, the exact mechanism by which Tip60 is involved in the viral life cycle of KSHV needs further characterization, due in part to the multiple cellular functions of Tip60 (Tang et al., 2006; Sun et al., 2007; Coffey et al., 2012; Gao et al., 2014), as well as the incomplete understanding of KSHV-host interactions. Yet, as inhibition of Tip60 resulted in decreases in both lytic and latent gene expression, we consider it likely that Tip60 functions in multiple parts of the viral life cycle. For example, Lu et al. reported that LANA can become acetylated, and that this functions to regulate its repressive effects on lytic replication. As mentioned previously, Tip60 has been shown to bind to LANA. If LANA is acetylated during this interaction it may explain why we see decreased lytic gene expression associated with Tip60 inhibition. Furthermore, Tip60’s effect on KSHV replication could be attributed to its interaction with other viral proteins, such as ORF36, or interactions that have yet to be identified (Li et al., 2011). Tip60 contains a chromatin-binding domain that binds to methylated histones in order to facilitate histone acetylation and associated upregulation of transcription (Jeong et al., 2011; Kim et al., 2015). Thus, Tip60 could be directly recruited to the KSHV genome by recognizing certain histone epigenetic markers. Therefore, Tip60’s effect on KSHV could be due to many possibilities, likely in combination. For example, Tip60 may be responsible for both the acetylation of LANA as described above, as well as upregulating transcription through epigenetic modifications at either the latency or kaposin promoter. Additionally, it is worth noting that we did not observe a decrease in latent gene transcription in Tip60 inhibitor treated BC-3 cells. However, both BCBL-1 and BC-3 cells exhibited decreased cell viability following long-term treatment with Tip60 inhibitors. It is also notable that while Tip60 inhibitor treatment resulted in decreased lytic gene expression in induced BC-3 cells (Supplementary Figure S3), this decrease in lytic genes was not as significant as that observed in the BCBL-1 cells (**Figure 3**). Thus, we postulate that there may be a difference between KSHV-infected cell lines with respect to initial susceptibility to Tip60 inhibition. This may be due to different levels in viral genome copies as well as genetic differences. Yet, it is important to note that we did observe decreased lytic gene expression, virion production, and cell viability following long term treatment in BC-3 cells; however, this effect was simply not as potent as the effect observed in BCBL-1.

Tip60 was initially discovered due to its ability to interact with HIV Tat protein (Kamine et al., 1996). Many studies since this discovery have shown that Tip60 is important for viral infection and oncogenesis of other viruses, including human T cell lymphotrophic virus (HTLV), human papilloma virus (HPV), and human cytomegalovirus (HCMV), in addition to g-herpesviruses (Awasthi et al., 2005; Li et al., 2011; Reitsma et al., 2011; Shamay et al., 2012; Hong et al., 2015). Tip60 dysregulation has also been linked to a set of cancers; nuclear Tip60 levels are decreased in breast carcinomas (Gorrini et al., 2007). Conversely, following androgen depletion, the nuclear levels of Tip60 increase in prostate cancers (Halkidou et al., 2003; Shiota et al., 2010). As such Tip60 inhibitors have been tested previously for their anticancer effects. Coffey et al. (2012) described that treatment of prostate cancer cells with NU9056 induces cell apoptosis through activation of caspases in a time-dependent manner. Given that Tip60 regulates KSHV latent gene expression that contributes to KSHV oncogenesis, we tested the feasibility of Tip60 inhibitors for treating KSHV-positive lymphomas. Following long-term treatment with Tip60 inhibitors, the cell viability of KSHV-infected B lymphoma cell lines (BCBL-1, BC-3, and MC116.219) was much lower than that of KSHV-naive cell lines (BJAB and MC116). Particularly, MC116.219 and MC116 cells showed different responses to Tip60 inhibitors (**Figure 4E**). MC116.219 cells are derived from parental MC116 cells (Dollery et al., 2014), so these cells share a very similar genetic background, with the exception that MC116.219 cells harbor the KSHV viral genome. Therefore, we postulate that KSHV infection increases the susceptibility of cancer cells to Tip60 inhibitors.

Combined with our other results, which showed that Tip60 promotes KSHV lytic and latent gene expression, we believe that it will be promising to treat KSHV-associated cancers with Tip60 inhibitors. However, we have only tested the effect of Tip60 inhibitors on KSHV-positive PEL cells; thus, it would be advantageous to also examine the effect of Tip60 inhibitors on KS, which remains the second most common cancer in HIV-infected patients worldwide (Gbabe et al., 2014). Unfortunately, this could be difficult, as presently there are no KS cell lines and no suitable tumor models for this disease (Sturzl et al., 2013). Lastly, it will be important to investigate if Tip60 inhibitors can inhibit KSHV-positive tumors through other mechanisms that are independent of KSHV viruses.

## Author Contributions

JZ and NS conceived the project. JZ, NS, and SS designed the study, analyzed the results, and wrote the paper. SS performed the experiments. GF, MJ, MD, JM, SJ, PD, and MS provided reagents and advised the study. All authors reviewed results and approved the final version of this manuscript.

## Conflict of Interest Statement

The authors declare that the research was conducted in the absence of any commercial or financial relationships that could be construed as a potential conflict of interest.
