# Supplementary material for: Inhibition of Tip60 Reduces Lytic and Latent Gene Expression of Kaposi’s Sarcoma-Associated Herpes Virus (KSHV) and Proliferation of KSHV-Infected Tumor Cells
Source: Front Microbiol. 2018 Apr 24;9:788. doi: 10.3389/fmicb.2018.00788 (PMC5928232; doi:10.3389/fmicb.2018.00788)
Supplement: Supplementary file 1 [file Data_Sheet_1.docx]

Supplementary Material

Inhibition of Tip60 Reduces Lytic and Latent gene expression of Kaposi’s Sarcoma-associated Herpes Virus (KSHV) and Proliferation of KSHV-infected Tumor Cells

**Sydney Simpson^1^, Guillaume Fiches^1^, Maxime J. Jean^1^, Michael Dieringer^1^, James McGuinness^1^, Sinu P. John^2^, Meir Shamay^3^, Prashant Desai^4^, Netty Santoso^1,*^, Jian Zhu^5,*^**

*** Correspondence:** Jian Zhu: Jian.Zhu@osumc.edu ; Netty Santoso: Netty Santoso@urmc.rochester.edu

## Supplementary Figures


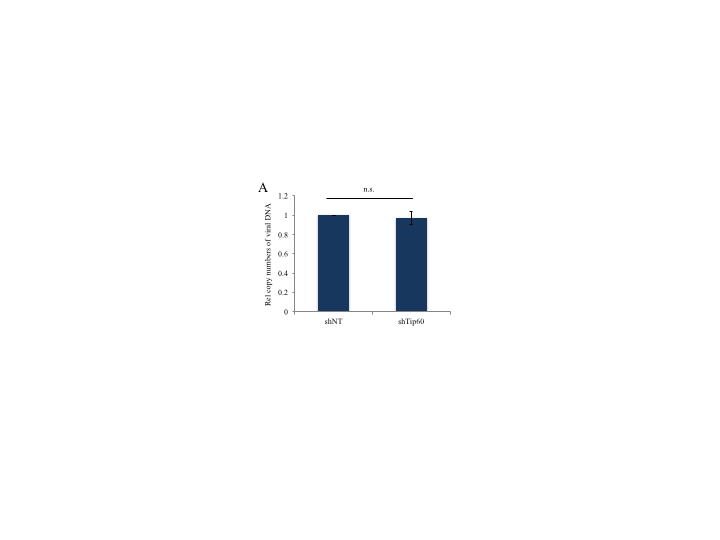


**Figure S1. Knockdown of Tip60 in HEK293T.Bac36 cells does not affect viral genome copy numbers.** DNA was extracted from HEK293T.Bac36 cells stably expressing either shNT or shTip60. Viral genome copy number was quantified using RT-qPCR, probing for LANA. Numbers are normalized to shNT. Values were normalized to GAPDH. The results from three independent experiments are represented as mean+ s.e.m. ** = p < 0.05,* *** = p < 0.01, student t-test*.


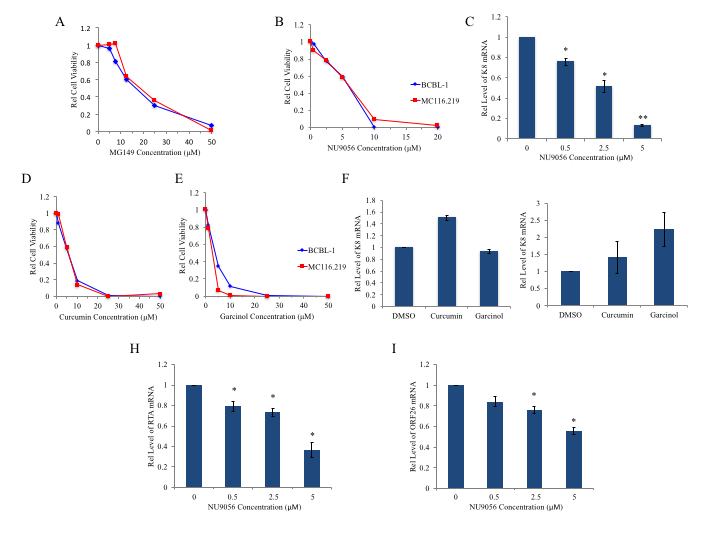
 **Figure S2. HAT inhibitor cell viability titrations and effects on MC116.219 cell reactivation** A, B) BCBL-1 (blue) and MC116.219 cells (red) were treated with Tip60 inhibitors, MG149 (A) or NU9056 (B), at a series of concentration for 24 hours. Cell viability was measured and normalized to DMSO-treated cells. C) MC116.219 cells were pre-treated with NU9056 at a set of concentrations (0.5, 2.5, 5 µM) for 6 hours, prior to incubation with TPA/SB for 48 hours to induce KSHV reactivation. mRNA level of KSHV K8 lytic gene was measured by RT-qPCR, and normalized to DMSO-treated cells. GAPDH mRNA was also measured as a loading control. C, D) BCBL-1 (blue) and MC116.219 cells (red) were treated with were treated with curcumin (D) or garcinol (E), at a series of concentration for 24 hours. Cell viability was measured and normalized to DMSO-treated cells. F,G) BCBL-1 (F) and MC116.219 (G) cells were pre-treated with curcumin (1 µM) or garcinol (0.5 µM) for 6 hours, prior to incubation with TPA/SB for 48 hours to induce KSHV reactivation. mRNA level of KSHV K8 lytic gene was measured by RT-qPCR, and normalized to DMSO-treated cells. H) MC116.219 cells were pretreated with with Tip60 inhibitors or DMSO then stimulated with TPA/SB as before. RTA mRNA and I) ORF26 levels were measured by RT-qPCR. RT-qPCR values were normalized to GAPDH mRNA. The results from three independent experiments are represented as mean + s.e.m. ** = p < 0.05,* *** = p < 0.01, student t-test*, n.s. = non-specific.

**
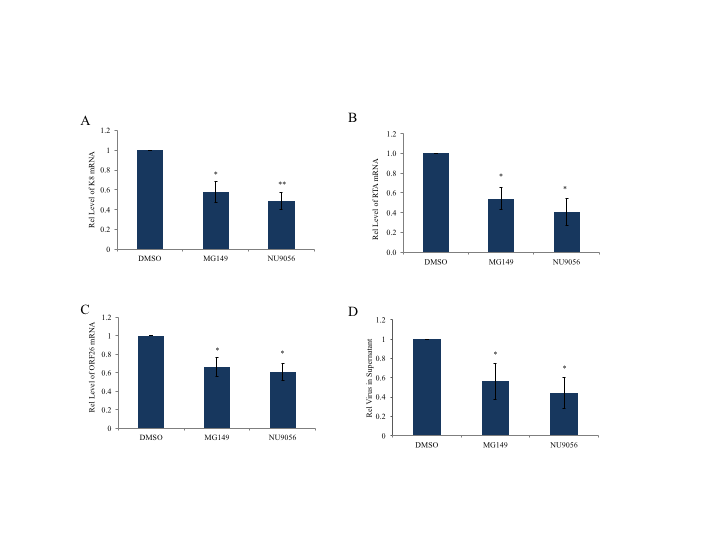
Figure S3. Inhibition of Tip60 decreases lytic gene transcripts and virus production in BC-3 cells.** A) BC-3 cells were pretreated with Tip60 inhibitors MG149, NU9056 or DMSO (control) for 6 hours prior to incubation with TPA/SB. After 48 hours cells were lysed and mRNA levels of K8, B) RTA and C) ORF26 were analyzed. D) BC-3 cells were stimulated as before, then cell-free supernatant was harvested after 72 hours. RT-qPCR measuring LANA was performed to measure the relative amount of virus in the cell-free supernatant. RT-qPCR values were normalized to GAPDH mRNA. The results from three independent experiments are represented as mean + s.e.m. ** = p < 0.05,* *** = p < 0.01, student t-test*, n.s. = non-specific.
