# Supplementary material for: Inhibition of Tip60 Reduces Lytic and Latent Gene Expression of Kaposi’s Sarcoma-Associated Herpes Virus (KSHV) and Proliferation of KSHV-Infected Tumor Cells
Source: Front Microbiol. 2018 Apr 24;9:788. doi: 10.3389/fmicb.2018.00788 (PMC5928232; doi:10.3389/fmicb.2018.00788)
Supplement: Supplementary file 2 [file Data_Sheet_2.docx]

Supplementary Material

Inhibition of Tip60 Reduces Lytic and Latent gene expression of Kaposi’s Sarcoma-associated Herpes Virus (KSHV) and Proliferation of KSHV-infected Tumor Cells

**Sydney Simpson^1^, Guillaume Fiches^1^, Maxime J. Jean^1^, Michael Dieringer^1^, James McGuinness^1^, Sinu P. John^2^, Meir Shamay^3^, Prashant Desai^4^, Netty Santoso^1,*^, Jian Zhu^5,*^**

*** Correspondence:** Jian Zhu: Jian.Zhu@osumc.edu ; Netty Santoso: Netty Santoso@urmc.rochester.edu

## Original Immunoblot Images

**
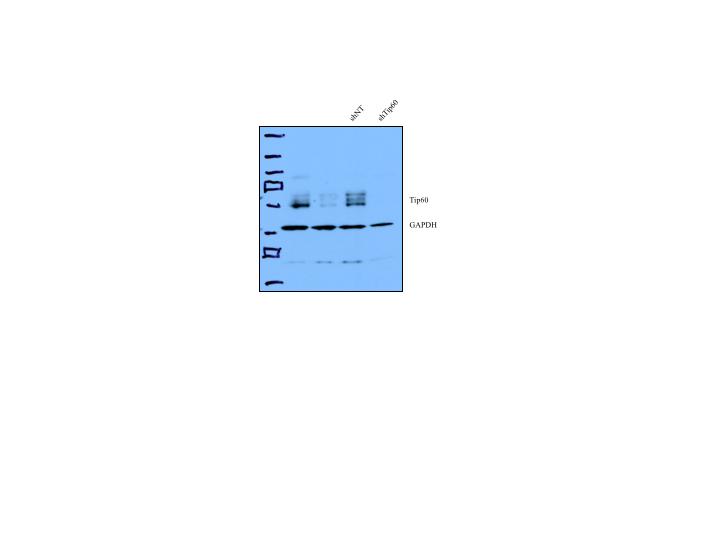
**

**Figure 1A original immunoblot**


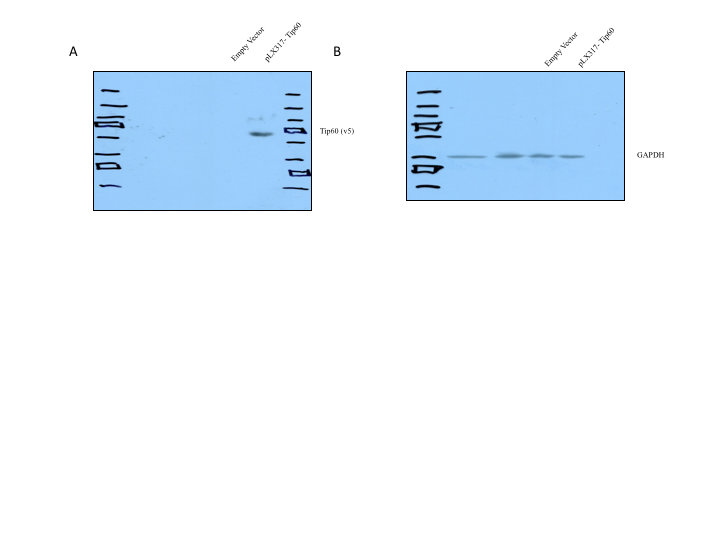

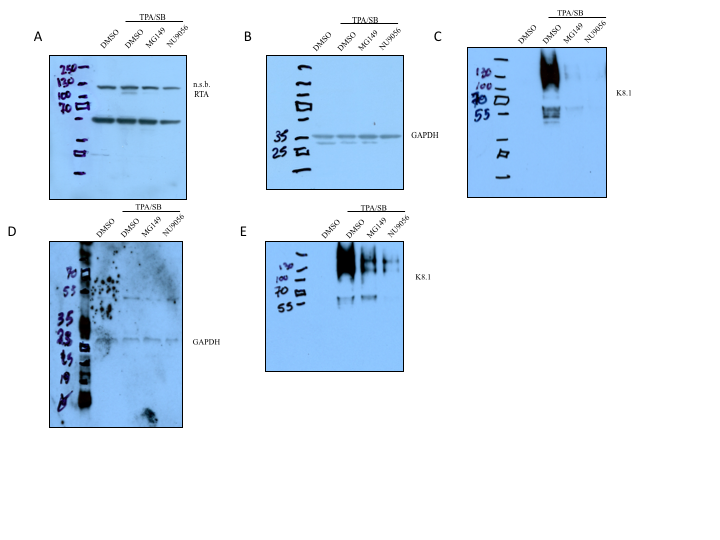
**Figure 2A Original Immunoblots for A) anti-V5 (Tip60) and B) GAPDH**

**Figure 3B Original Immunoblots for A) RTA and B) GAPDH. C&D) Figure 3E Original Immunoblots for C) K8.1 and D) GAPDH. E) Original Figure 3F immunoblot for K8.1**


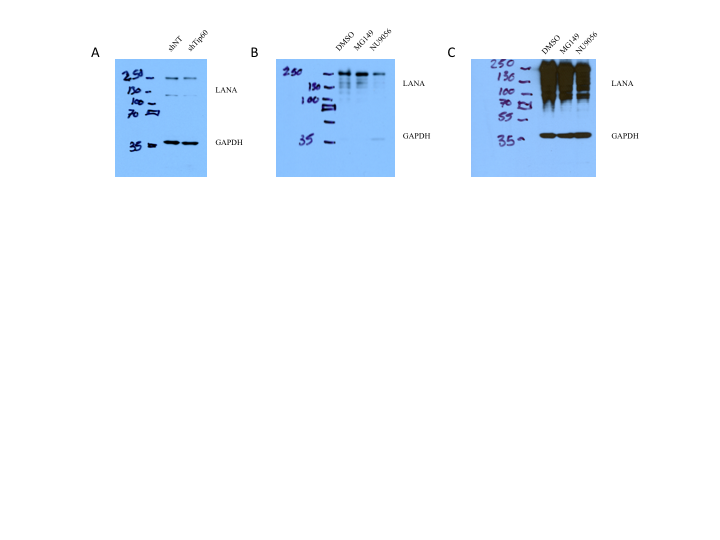
**A) Figure 4B original LANA and GAPDH immunoblot. B&C) Figure 4D original B) LANA and C) 4D original B)LANA and C) GAPDH immunoblots.**
